# Moderators and mediators of pedometer use and step count increase in the "10,000 Steps Ghent" intervention

**DOI:** 10.1186/1479-5868-6-3

**Published:** 2009-01-12

**Authors:** Katrien De Cocker, Ilse De Bourdeaudhuij, Wendy Brown, Greet Cardon

**Affiliations:** 1 Department of Movement and Sports Sciences, Ghent University, Ghent, Belgium; 2 School of Human Movement Studies, University of Queensland, Brisbane, Australia

## Abstract

**Background:**

The European pedometer-based "10,000 Steps Ghent" whole community intervention for 228,000 residents was found to be effective in increasing step counts by an average of 896 steps/day in a sub-sample of adults. The present study aimed to examine the characteristics of intervention participants (n = 438) who (1) used a pedometer and (2) increased their step counts. Additionally, the third aim was to examine the mediational effect of pedometer use on step count change.

**Methods:**

The study sample consisted of 438 adults (207 male, mean age 49.8 (13.1) years). Binary logistic regressions were used to examine whether individual characteristics (gender, age, educational level, employment status, self-reported health condition, baseline step counts, baseline sitting time, baseline transport-related PA) and intervention exposure variables (having heard/seen a PA promotion message, being aware of the PA guidelines, and knowing about "10,000 Steps Ghent") were associated with (1) pedometer use and (2) a step count increase of 896 steps/day or more. Using pooled data (n = 864) from the intervention and comparison participants, a mediation analysis was conducted to see if the change in step counts was mediated by pedometer use.

**Results:**

Age (49 years or more: OR = 3.19, p < 0.005), awareness of a PA promotion message (OR = 2.62, p < 0.01) and awareness of "10,000 Steps Ghent" (OR = 2.11, p < 0.05) were significantly associated with pedometer use. Participants with a college or university degree (OR = 1.55, p < 0.05) and those who used a pedometer (OR = 2.06, p < 0.05) were more likely to increase their steps by 896 steps/day or more. This increase was less likely among those with baseline step counts above 10,000 steps/day (OR = 0.38, p < 0.001). The mediation analysis revealed that pedometer use partly mediated step count change.

**Conclusion:**

Pedometer use was more likely in older participants and in those who were aware of the "10,000 Steps" campaign. Increasing step counts was more likely among those with higher education, baseline step counts below 10,000 steps/day and those who used a pedometer. Pedometer use only partly mediated the intervention effect on step counts.

## Background

Low levels of physical activity (PA) are associated with an increased risk for adverse physiological and mental health outcomes including cardiovascular diseases, obesity, hypertension, diabetes mellitus type 2, different types of cancer, osteoporosis, and depression and anxiety [1]. Therefore, international guidelines recommend that all healthy adults aged 18–65 should engage in moderate-intensity aerobic PA for a minimum of 30 minutes on five days each week, or in vigorous-intensity aerobic PA for a minimum of 20 minutes on three days a week [2]. Nevertheless, the majority of American (60%) [1], Australian (43%) [3], and European (43–87%) [4] adults do not meet this recommendation. Consequently, diverse interventions in various settings and specific populations have been developed and implemented to promote PA [5].

Pedometers, which objectively measure ambulatory activities throughout the day in the form of step counts, have become popular as monitoring and motivational tools in PA interventions. Pedometers are easy to use and relatively inexpensive compared with other motion sensors (pedometer: approximately US $ 20–50; accelerometer: approximately US $ 150–500). Evidence suggests that the use of pedometers is associated with significant increases in PA levels [6,7] and significant improvements in health outcomes among adults [6]. In addition, step count goals such as '10,000 steps/day' have been used in the promotion of PA [8].

Pedometer interventions appear to be effective both in smaller settings (e.g. workplaces [9,10], churches [11], primary care [12,13]), and in whole community-based trials (e.g. "The Step-by-Step Trial" [7], "10,000 Steps Rockhampton" [14], "10,000 Steps Ghent" [15], and "Canada on the Move" [16]). The Australian "Step-by-Step Trial" showed that pedometer use can enhance the effects of a self-help walking program. The main outcome of the "10,000 Steps Rockhampton" intervention was that the downward trend in the percentage of citizens classified as active in the comparison community was not evident in the intervention community [14]. The "10,000 Steps Ghent" whole community intervention succeeded in increasing step counts (average step count increase of 896 steps/day, p < 0.001) after one year of intervention [15]. Despite the overall effectiveness of these community-based interventions, it is possible that they only reached people who were already active, or that the intervention was more efficient for isolated subgroups (e.g. 20–30 year olds). In the "10,000 Steps Rockhampton" project, women were the early adopters of pedometer use; people aged 45 or more, those with higher levels of education, employed people, and those with an 'obese' BMI were more likely to report using a pedometer [14,17]. The results of the "Canada on the Move" project also showed that pedometer use was more likely among women and older people (44–64 years) [16].

Data from the "10,000 Steps Ghent" intervention provide an opportunity to examine whether the characteristics of people who used a pedometer and increased their step counts in this European whole community intervention, were similar to those seen in Australia and Canada. During the multi-strategy intervention, pedometer use was promoted in Ghent at different locations: pedometers could be bought or borrowed at the participants' own discretion. The first aim of the present study was to examine whether self-selected pedometer use in the intervention sample was associated with individual characteristics (gender, age, education, employment status, self-reported health, baseline step counts, baseline sitting time, and baseline transport-related PA) and intervention exposure variables (hearing or seeing a PA promotion message, knowing the amount of PA required for health benefit, and knowing about "10,000 Steps Ghent"). The second aim was to compare the individual characteristics and intervention exposure variables in intervention participants who increased their step counts considerably and those who did not. Using pooled data from the intervention and comparison participants, the third aim was to examine the potential mediating effect of pedometer use on step count change.

## Methods

### Procedures

Prior to the "10,000 Steps Ghent" whole community intervention for 228,000 residents, 2081 randomly selected 25–75 year old adults, living in the city of Ghent (Belgium), were invited to participate. Of those, 872 were interested, 648 completed baseline measurements and 440 participated in the one year follow-up. Baseline and follow-up measures consisted of the completion of the telephone-administered long version of the International Physical Activity Questionnaire (IPAQ) and the self-monitoring of pedometer steps for 7 consecutive days, using a daily activity log. At follow-up, participants were asked to also complete a questionnaire relating to awareness of the "10,000 Steps Ghent" project. Participants had to return the pedometer after baseline and follow-up data collection. Details of the study procedures have been described previously [15].

### Participants

For aims 1 and 2, the sample consisted of 438 intervention participants (207 male) with a mean age of 49.8 (13.1) years. About 52.9% (n = 232) had a college or university degree and 67.5% (n = 295) were employed. The majority (n = 344, 79.1%) reported good to excellent health (see Additional file 1). At baseline, the sample took an average of 9595 (4256) steps/day, spent 20 (27) minutes/day in transport-related PA and 396 (164) minutes/day sitting. For aim 3, pooled data from these 438 intervention participants and from 426 participants from the comparison city of Aalst [15] were used. All participants provided written informed consent and the study was approved by the Ethical Committee of Ghent University.

### "10,000 Steps Ghent" intervention

During the intervention, PA was promoted in the entire city of Ghent, using the central theme of '10,000 steps/day', with secondary taglines of 'every step counts' (elke stap telt) and 'every revolution (of bicycle pedals) counts' (elke trap telt). The guidelines, recommending 30 minutes of moderate-intensity PA on five days a week, or 20 minutes vigorous-intensity PA on three days a week [2], were also promoted. Multiple strategies, based on the social ecological model, were designed to intervene at the individual, social and environmental level. A local media campaign (street signs, press conferences, advertisements), the sale and loan of pedometers, the use of a website, workplace projects, projects for older people and the dissemination of information through health professionals, schools and associations were concurrently implemented. More details on the intervention strategies can be found elsewhere [15].

### Instruments

#### IPAQ

The long version of the IPAQ was used to assess sitting and PA in different domains (work, transport, household and leisure time) in a usual week. The IPAQ is known to be a valid and reliable instrument for assessing PA in Europe [18] and Belgium [19]. In the present study, only sitting time and transport-related PA items were used, since walking and biking for transport reflect the concept of lifestyle PA promoted through "10,000 Steps Ghent".

#### Pedometer

The valid, accurate, and reliable Yamax Digiwalker SW-200 (Yamax Cooperation, Tokyo, Japan) was used in the present study to measure free-living step counts [20].

#### Activity log

Participants were asked to record the date, steps taken at the end of each day, and the type and duration of non-ambulatory activities (i.e. biking and swimming). For every minute of reported biking and/or swimming, researchers added 150 steps to the daily total number of reported step counts [15,21]

#### Questionnaire relating to awareness of the project

The following questions, asked at one-year follow-up, were used in the present study: Have you heard or seen any messages about PA promotion? (yes/no); Do you have any idea about the amount of PA that is required for health benefit? (yes/no + open ended); Have you heard of the "10,000 Steps Ghent" project? (yes/no); Have you used a pedometer in the last 10 months? (yes/no). A timeframe of 10 months was used to avoid inclusion of baseline pedometer measurements.

### Data Analysis

All individual characteristics (gender, age, education, employment status, self-reported health, baseline step counts, baseline sitting time, and baseline transport-related PA) and intervention exposure variables (hearing or seeing a PA promotion message, knowing the PA guidelines, knowing about "10,000 Steps Ghent", and self-selected pedometer use during the intervention) were interpreted as categorical variables and dichotomized using median scores as follows: age (M = 49 year), baseline sitting time (M = 6.2 hours/day) and baseline transport-related PA (M = 10.7 minutes/day). Descriptive statistics (numbers and percentages) were calculated using cross tabs. Binary logistic regression was used to examine whether individual characteristics and intervention exposure variables were associated with (1) pedometer use during the intervention and (2) greater than mean step count increase (> 896 steps/day). Results are expressed as odds ratios with 95% confidence intervals and p values. All data were analyzed using SPSS 15.0 for Windows (SPSS Inc., Chicago, USA) and statistical significance was set at 0.05.

**Figure 1:**
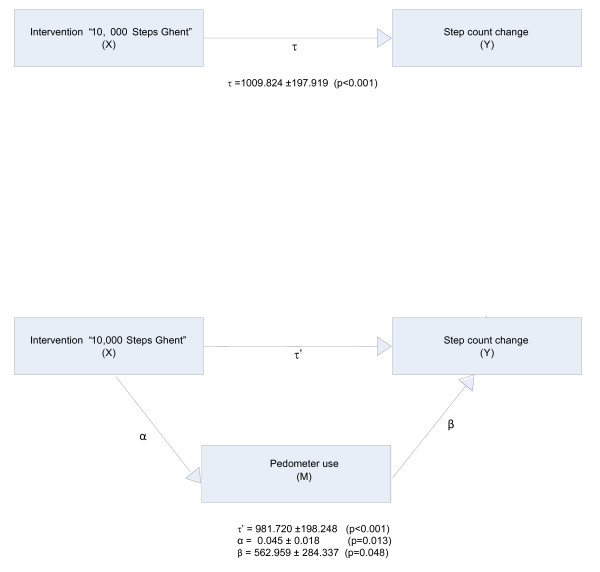
**Path diagram for the analysis of the mediational effect of pedometer use on step count change**.

As suggested by Cerin et al [22], the Freedman-Schatzkin difference-in-coefficients test was used to assess the mediational effect of pedometer use on step count change. A measure of step count change between baseline and one year follow-up, free of auto correlated error, was recreated by regressing the step counts at follow-up onto the step counts at baseline. The Freedman-Schatzkin test assesses a mediational effect by comparing the relationship between the independent variable (the intervention) and the dependent variable (step count change) before and after adjustment for the mediator (pedometer use) (see figure 1). It tests the null hypothesis that the difference between the unadjusted (without pedometer use as mediator: τ) and adjusted (with pedometer use as mediator: τ') regression coefficients of the independent variable is zero. The test consists of two regression analyses. The first examines the impact of the intervention condition (dummy variable) on the residualized change step count scores, providing an estimate for τ (relationship between intervention condition and step count change before adjusting for the mediator). The second looks at the effect of the intervention on residualized change step count scores after controlling for pedometer use (dummy variable), giving an estimate for τ' which represents the independent effect of the intervention condition on step count change after adjusting for the mediator. The significance test of the mediational effect is computed by dividing (τ - τ') by its standard error and comparing the obtained value to a t-distribution with N – 2 degrees of freedom [22]. If the t-value is > 1.984 there is a significant mediation effect at the 5% level. The proportion of the intervention effect mediated by pedometer use was calculated by subtracting the adjusted relationship between the intervention exposure and step count change (τ) from the unadjusted relationship (τ'), and dividing the sum by the unadjusted value ((τ - τ')/τ) [23].

## Results

Only 72 (16.4%) intervention participants used a pedometer during the one-year intervention period. Participants older than 49 years (p = 0.001), those who reported having heard or seen a message about PA promotion (p = 0.006), and those who knew about "10,000 Steps Ghent" (p = 0.047) were more likely to report pedometer use. None of the other potential explanatory variables was significantly associated with pedometer use during the intervention (see Additional file 1). Post hoc chi-square analyses examined the inter-relationship between these significant moderating variables. There was a strong positive relationship between PA promotion message recall and project awareness (χ^2 ^= 58.7, p < 0.001). About 44.2% of those who reported having heard or seen a PA promotion message (χ^2 ^= 7.3, p = 0.007), and 46.5% of those aware of the project (χ^2 ^= 3.2, p = 0.074), were older than 49 years.

Overall, 209 (47.5%) participants showed an increase in average step counts of 896 steps/day or more at one-year follow-up. Participants with a college or university degree (p = 0.046), and those who used a pedometer during the intervention (p = 0.014) were more likely to have increased their step counts by 896 steps/day or more, while those with a baseline average step count level of more than 10,000 steps/day were less likely to have increased their step counts by 896 steps/day or more (p < 0.001). None of the remaining variables was significantly associated with the step count increase of 896 steps/day or more (see Additional file 1). Post hoc analyses revealed that more than half those with a college or university degree (54.8%), had fewer than 10,000 steps/day at baseline (χ^2 ^= 3.8, p = 0.051).

Regression coefficients of the different paths are shown in Figure 1. Results revealed that the intervention "10,000 Steps Ghent" was a significant predictor of step count change (p < 0.001) before adjusting for the mediator, pedometer use. After adjusting for pedometer use, the intervention condition remains a significant predictor of step count change (p < 0.001), however the value of the adjusted regression coefficient (τ') is significantly lower than the regression coefficient before adjusting for pedometer use (τ) (t = 2.1), pointing to a significant mediation effect. This analysis revealed that pedometer use partly mediated ((τ - τ')/τ = 0.028 or 2.8%) the effect of the intervention on step count change.

## Discussion

The "10,000 Steps Ghent" whole community intervention was effective in increasing step counts: almost half the intervention participants increased their step counts on average by 896 steps/day or more at one-year follow-up. However, the proportion of intervention participants using a pedometer through loan or sale service during the intervention was modest (16%). The purpose of the present study was to investigate which individual characteristics and exposure variables were associated with pedometer use and step count increase.

Pedometer use in Ghent (16%) was remarkably similar to that in the Australian "10,000 Steps" intervention in Rockhampton (18%) [17]. The predictors of pedometer use were also very similar in these two studies and in the "Canada on the Move" intervention. For example, in all three studies older people (over 49 in Ghent, over 45 in Rockhampton [17], and in the 44–64 year age group in Canada [16]) were more likely to have used a pedometer. Other previous pedometer-based studies have shown that PA is inversely associated with age [24-26], so the finding that pedometer use is more prevalent among older people is encouraging. Furthermore, in all three studies, individuals being exposed to program variables (having heard or seen a message about PA promotion and knowing about the "10,000 Steps" project in Ghent; having seen the street signage/walking trials and visited the website in Rockhampton [17]; and campaign awareness in Canada [16]) were more likely to report using a pedometer. The latter seems a logical finding, however explaining why older participants were more likely to use a pedometer is difficult, as participants who were aware of PA promotion messages or the "10,000 Steps Ghent" project, were no more likely to be older than 49 years than those who were unaware. It is possible that older people had more time or more interest in trying out a pedometer. Mostly, they care for their health, and like to have defined guidelines and goals concerning their health behavior. Using a pedometer gives them the opportunity to set and reach goals regarding their PA.

Although pedometer use was more likely among women, and employed and higher educated individuals in Rockhampton [17], and more likely among women, college and university graduates, and high-income earners in Canada [16], gender, education and employment status were not significantly associated with pedometer use in the present Ghent study.

Participants with a college or university degree were however more likely to record a step count increase in this project, suggesting that more efforts are needed to reach those with lower levels of education. A previous cross-sectional pedometer study conducted in the United States, also revealed that higher educated individuals had significantly more daily step counts than lower educated persons [24]. Wyatt et al [25] on the other hand, found that steps did not differ significantly as a function of education level in Colorado. However, in the present study, there was a tendency for those with higher education to have a baseline step count below 10,000 steps/day. Furthermore, the present findings showed that less active individuals (i.e. those with a baseline step count level below 10,000 steps/day, and consequently those with a college or university degree) were more likely to increase their steps. This promising outcome suggests that the whole-community intervention, which was designed to reach sedentary people, was indeed effective for those most in need of (more) PA, and not for already active individuals.

As pedometer use was only one of the strategies promoted during this multi-component intervention, it was interesting to find that it was associated with the observed increase in step counts. Our analyses showed that the multi-strategy intervention was successful in promoting and stimulating pedometer use, which in turn resulted in positive step count changes. The intervention effect was however only partly mediated by pedometer use (2,8%), which is not surprising as only one in six people reported using a pedometer. The findings suggest that, although the pedometer was valuable in promoting increased step counts in a whole community, the other strategies (the media campaign, street signs, website, workplace projects, working with health professionals and targeting older people) could also be important. Post hoc mediation analyses showed that the intervention was significantly mediated by awareness through street signs (11.1% mediation) and workplace projects (7.3% mediation). This suggests that promotion, available for the whole community (i.e. street signs), or in smaller settings (i.e. workplaces) has an effect on step count change. The latter mediating effects were even greater than that of pedometer use, which was thought to be an important mediator of the intervention. Notwithstanding, this is the first time that the mediating effect of pedometer use on increasing activity has been demonstrated in a whole community intervention.

One limitation of this study is that the questions about pedometer use were not asked at baseline, so information on pre-intervention pedometer use was not available. However, a previous Belgian study [27] has shown that pedometer use was not common in East-Flanders at the time that this intervention was implemented. A second limitation is the lack of information on BMI or other health variables, and additional socio-demographic variables like marital status, income and job classification. The strengths of this study include the large sample size, the random sample of participants for the evaluation (i.e. they were not 'volunteers' in the intervention program), the age range (25–75 years), and the longitudinal step count data.

## Conclusion

The findings suggest that age and intervention awareness were positively associated with pedometer use, which was in turn (along with education and low baseline step counts) a significant predictor of step count increase in this whole community intervention. Mediation analyses showed that the intervention effect on increasing step counts was only partly mediated by pedometer use, illustrating the importance of the other strategies used in this campaign.

## Competing interests

The authors declare that they have no competing interests.

## Authors' contributions

KDC participated in the design of the study, collected and analyzed the data, and led the writing of the paper. KDC wrote the manuscript, while IDB, WB and GC participated in the design of the study, collaborated with partners on the development of the intervention, discussed the analysis plan, and provided substantive feedback on the manuscript. All authors have read and approved the final manuscript.

## Supplementary Material

Additional file 1 **Table 1: Analysis of the moderator effects on pedometer use and step count increase.** Click here for file
