# Supplementary material for: Moderators and mediators of pedometer use and step count increase in the "10,000 Steps Ghent" intervention
Source: Int J Behav Nutr Phys Act. 2009 Jan 12;6:3. doi: 10.1186/1479-5868-6-3 (PMC2628637; doi:10.1186/1479-5868-6-3)
Supplement: Additional file 1 — Table 1: Analysis of the moderator effects on pedometer use and step count increase. [file 1479-5868-6-3-S1.doc]

Table 1: Analysis of the moderator effects on pedometer use and step count increase.

**Used PM Increased step counts ≥ 896**

**N N using PM (%) adj OR 95% CI P N N ↑ SC (%) adj OR 95% CI P**

Gender

Male 209 25 (12.0) 1.00^c^ 207 91 (44.0) 1.00 ^c^

Female 231 47 (20.3) 1.48 0.82-2.65 0.191 231 118 (51.1) 1.23 0.81-1.85 0.336

Age

< 49 years 219 26 (11.9) 1.00 ^c^ 218 109 (50.0) 1.00 ^c^

≥ 49 years 219 45 (20.5) **3.19** **1.65-6.19 0.001** 218 99 (45.4) 0.73 0.44-1.20 0.217

Educational level

High school 207 35 (16.9) 1.00 ^c^ 206 87 (42.2) 1.00 ^c^

College/university 233 37 (15.9) 0.85 0.46-1.56 0.595 232 112 (52.6) **1.55 1.01-2.40 0.046**

Employment status

Unemployed 144 25 (17.4) 1.00 ^c^ 143 67 (46.9) 1.00 ^c^

Employed 296 47 (15.9) 1.10 0.54-2.22 0.802 295 142 (48.1) 1.03 0.60-1.76 0.924

Health condition

Weak-moderate 91 15 (16.5) 1.00 ^c^ 91 40 (44.0) 1.00 ^c^

Good-excellent 346 56 (16.2) 1.12 0.56-2.22 0.749 344 167 (48.5) 1.16 0.71-1.88 0.560

Baseline average daily step counts

< 10,000 251 34 (13.5) 1.00 ^c^ 251 140 (55.8) 1.00 ^c^

≥ 10,000 187 38 (20.3) 1.44 0.78-2.64 0.245 187 69 (36.9) **0.38 0.24-0.60 <0.001**

Baseline daily sitting time

< 6.2 hours 228 41 (18.0) 1.00 ^c^ 227 108 (47.6) 1.00 ^c^

≥ 6.2 hours 210 31 (14.8) 1.12 0.61-2.07 0.709 209 101 (48.3) 0.82 0.53-1.27 0.372

Baseline daily transport-related PA

< 10.7 minutes 223 30 (13.5) 1.00 ^c^ 223 112 (50.2) 1.00 ^c^

≥ 10.7 minutes 214 41 (19.2) 1.25 0.71-2.20 0.446 212 96 (45.3) 0.92 0.61-1.38 0.676

Having heard/seen any message about PA promotion

No 188 16 (8.5) 1.00 ^c^ 187 83 (44.4) 1.00 ^c^

Yes 251 56 (22.3) **2.62** **1.33-5.19 0.006** 250 126 (50.4) 1.17 0.75-1.82 0.489

Knowing the amount of PA required for health benefit

No 231 25 (10.8) 1.00 ^c^ 231 111 (48.1) 1.00 ^c^

Yes 209 47 (22.5) 1.86 0.96-3.60 0.064 207 98 (47.3) 0.86 0.54-1.38 0.538

Knowing about ‘10.000 Steps Ghent’

No 149 11 (7.4) 1.00 ^c^ 148 70 (47.3) 1.00 ^c^

Yes 291 61 (21.0) **2.11** **1.01-4.40 0.047** 290 139 (47.9) 1.02 0.65-1.61 0.934

Using a PM during the intervention

No 366 166 (45.4) 1.00 ^c^

Yes 72 43 (59.7) **2.06 1.15-3.67 0.014**

PM = pedometer

Adj OR = adjusted odds radio

CI = confidence interval

↑= increasing

SC = step counts

^c^ reference group

PA = physical activity
